# Continuous renal replacement therapy in critically ill children: single-center experience

**DOI:** 10.3906/sag-2006-227

**Published:** 2021-02-26

**Authors:** Hatice ERKOL TUNCER, Mesiha EKİM, Emel OKULU, Begüm ATASAY, Tanıl KENDİRLİ

**Affiliations:** 1 Department of Pediatrics, Faculty of Medicine, Ankara University, Ankara Turkey; 2 Department of Pediatric Nephrology, Faculty of Medicine, Ankara University, Ankara Turkey; 3 Department of Neonatology, Faculty of Medicine, Ankara University, Ankara Turkey; 4 Department of Pediatric Intensive Care Unit, Faculty of Medicine, Ankara University, Ankara Turkey

**Keywords:** Continuous renal replacement therapy, acute kidney injury, critically ill children, fluid overload, hemodiafiltration, hemofiltration

## Abstract

**Background/aim:**

Continuous renal replacement therapy (CRRT) has significant benefits in the treatment of critically ill children.The objective of this study is to describe the treatment indications, methods, demographics, and outcome of the patients who received CRRT in our pediatric intensive care unit and neonatal care unit, and, according to these results, we also aimto make improvements in our unit-based interventions.

**Materials and methods:**

In this single-centered study, we retrospectively evaluated medical charts of the patients admitted to our intensive care units and received CRRT between February 2010 and November 2015.

**Results:**

Fifty of 60 patients were included in this study. Newborns made up 28% (n = 14) of the patients. The mean body weight was 18.4 kg (2.3-98 kg). CRRT indications were fluid overload (30%), acute kidney injury (40%), metabolic disease (24%), electrolyte impairment (4%), and drug intoxication (2%). The most common method of CRRT was continuous venovenous hemodiafiltration (CVVHDF) (72%). The mean duration of CRRT was 135 hours (1-864) and totally 143 filters, polyarylethersulfon (n = 23.46%) and polyacrylonitrile (n = 27.54%) were used. Overall survival was 42%. The survival rate of newborns was significantly higher (P = 0.046).

**Conclusion:**

CRRT is a lifesaving method that can be applied to critically ill children with acute kidney injury and fluid overload at any age and weight by experienced teams.

## 1. Introduction

Acute kidney injury (AKI) is a common diagnosis in pediatric and neonatal intensive care units (PICU and NICU). It can be secondary to many conditions such as cardiac arrest, sepsis, shock states, heart failure, drug toxicity, acute hypoxic respiratory failure, cardiac surgery, multiorgan failure, liver disease. AKI is solely a factor for morbidity and mortality in critically ill children. Fluid overload (FO) is the biggest trouble in critically ill children when renal dysfunction develops. As FO is harmful to all organs especially cardiovascular and respiratory system, cardiovascular disorders, pulmonary edema, hypotension, and severe hypoxemia are seen when FO is over 10% [1-5]. Therefore, we must intervene AKI and FO. Some studies demonstrated that more than 10% of FO is an independent risk for prolonged mechanical ventilation and length of stay (LOS) in PICU, also for mortality [1]. Mortality rates are relatively high in pediatric renal failure, ranging from 66% to 90% in PICU [3].

Renal replacement therapies (RRT) in pediatric patients have developed rapidly since the 1980s, as an alternative to intermittent hemodialysis (IHD) which has disadvantages like cardiovascular instability, respiratory problems, catheter-related difficulties, and peritoneal dialysis (PD) having slow and low ultrafiltration efficiency. CRRT provided significant advantages in the treatment of critically ill patients [6]. The two most important factors affecting the choice of dialysis treatment are the indication of dialysis treatment and the clinical condition of the patient. The size of the molecules to be removed and the working principle of dialysis should be considered. The most important advantage of CRRT is a gradual solute and fluid excretion, mimicking renal functions and physiology, and helping to create conditions that will help the kidneys heal without compromising the hemodynamic balance [7,8]. In this study, we report our CRRT experiences about critically ill children in NICU and PICUs. 

## 2. Materials and methods

### 2.1. Collection and evaluation of data

We retrospectively evaluated the patients treated with CRRT and hospitalized in NICU and PICU of Ankara University Children’s hospital between February 2010 and November 2015. The ratio of total fluid intake to a fluid loss greater than 10% is defined as fluid overload. Also, the patient was oliguric/anuric and has pediatric risk, injury, failure, loss, end-stage renal disease (pRIFLE) score stage 3 or 4 [9] is defined as AKI. Another indication was the metabolic disease that is unresponsive to standard medical treatments and occurs in the presence of dialyzable toxic metabolic collection such as leucine, ammonium, refractory metabolic acidosis. The last group was severe poisoning with dialyzable toxic matter ingestion. 

Once there was an indication of CRRT, an appropriate size vascular access was provided, and then CRRT mode was determined. Priming was performed after establishing the suitable circuit for the patient’s BW (body weight) and anticoagulation was started to all patients. Patients’ demographic characteristics (age, sex), BW, primary disease, duration of hospitalization, The Pediatric Risk of Mortality (PRISM), and Pediatric Logistic Organ Dysfunction (PELOD) scores were recorded during admission.

Indications of CRRT, baseline biochemistry, (BUN, creatinine, sodium, potassium), and hourly urine output, based on the 24-h urine output were recorded before the onset of CRRT. The choice of CRRT modes, which are continuous venovenous hemodialysis CVVHD), continuous venovenous hemodiafiltration (CVVHDF), continuous venovenous hemofiltration (CVVH)], vascular access regions, CRRT initial settings, complications, other supportive modalities [Extracorporeal membrane oxygenation (ECMO), mechanical ventilation, plasma exchange], and the overall CRRT outcome were evaluated. Circuit and filter renewals, problems related to the machine, patient-related problems, factors related to clotting in the circuit were analyzed. We used to Gambro-Baxter (USA) dialysis machine and its proper filters. All patients were followed up with activated partial thromboplastin (APTT) time at appropriate intervals, and activated clotting time (ACT), which is a method that is easier and quicker, is applied additively to the patients who had a hemodynamic disorder and bleeding tendency.

The decision to terminate the CRRT is based on the recovery from the current problem like FO, AKI with good diuresis, insuperable issue, and deceased patients. Treatment success was defined as the ability to perform the planned CRRT program as long as the patient needed.

### 2.2. Statistical evaluation of data

SPSS 15.0 (SPSSInc., Chicago, IL, USA) package program was used in the evaluation of the data. Chi-square and/or Fisher’s exact test was used to compare categorical variables. Student’s T-test and/or one-way analysis of variance (ANOVA) was used for the continuous measurement values according to the number of categories. Analysis results were summarized by mean±standard deviation, median values, frequency distributions, and percentages, and P < 0.05 was considered statistically significant.

## 3. Results

Sixty children receiving CRRT in PICU and NICU between February 2010 and November 2015 were included in the study; however, 10 of them were excluded because of the missing data.

A total of 40% of all patients were female. Ages ranged from 3 days to 19 years; Nnwborns were constructed 28% (n = 14) of the group. The mean BW was 18.4 ± 20.1 kg (2.3-98 kg). The patients were further grouped as <5 kg, 5-10 kg, 10-20 kg, 20-50 kg and > 50 kg. The group with the highest number of patients was infants weighing less than 5 kg (n = 15, 30%).

Three of the 50 patients were still in hospital at the end of the study; therefore, the mean LOS in PICU/NICU for the remaining 47 patients was 13.1 ± 12.1 (1-47) days. PRISM and PELOD scores were 19 (6-45) and 20 (10-33), respectively. All the patients were received mechanical ventilator support, 8 (16%), and 12 (24%) of them were treated with ECMO and plasma exchange, respectively. The median PRISM score of the patients treated with ECMO was 28 (15-45) and the PELOD score was 32 (22-43). Laboratory findings before CRRT was; BUN 34 ± 33.9 (1-161) mg/dL, creatinine 1.35 ± 1.01 (0.08-4.6) mg/dL, sodium 138 ± 7.6 (124-158) mEq/L and potassium was 4.3 ± 1.8 (2-9.8) mEq/L. The 24-h urine output of the day before the onset of CRRT was 2.1 ± 2.8 (0-12.2) mL/kg/h.

Indication categories for CRRT are given in Table 1. The most common modality was CVVHDF (72%) and the least modality was CVVH (4%). 

**Table 1:** Indications of CRRT.

Indication	Patient (n)	%
Fluid overload	15	30
Acute kidney injury	20	40
Metabolic disease Urea Cycle defect (n = 6) Methylmalonic aciduria (n = 2) Maple syrup urine disease (n = 2) Lactic acidosis (n = 2)	12	24
Electrolyte impairment Hyperpotassemia (n = 2)	2	4
Drug intoxication Theophylline (n = 1)	1	2

Double-lumen hemodialysis catheters were placed into internal jugular vein (50%), femoral vein (36%), and subclavian vein (12%), and CRRT was applied from ECMO connection site in one patient. The median size of the catheter was 7 (5-11) F.

The mean duration of CRRT was 135 h (1-864), a sum of 143 filters [23 (HF20, 46%), polyarylethersulfon, 14 polyacrylonitriles (AN 69) (M60 28%), 13 M100 (26%)] were used. Some patients completed the treatment period with one circuit, while, in others, circuit replacement was required due to clot formation. The mean circuit lifespan was 39.1±27.5 (0.5-120) h. When we compared the patients having mean circuit lifespan >39.1 and <39.1 h, there was no difference in catheter sizes (P = 0.341) and circuit type (P = 0.369). However, 62.5% (n = 10) of 16 patients used a 9F catheter, the circuit lifespan was longer than the average. There was no significant relationship between catheter entry site and circuit lifespan (P = 0.537).

Priming was performed with saline (32%), 5% albumin (24%), and blood (44%) and heparin was used for anticoagulation in all patients and it was changed to citrate in bleeding patients. CRRT initial settings were recorded according to CRRT models and BW (Table 2). The most common side effect related to CRRT was hypotension (56%) (Table 3).

**Table 2:** CRRT initial settings by body weights.

Body weight(kg)	Blood flow rate(mL/kg/min)	Dialysate flow rate (mL/1,73 m²/h)	Replacement fluid flow rate (mL/kg/h)	Ultrafiltration rate (mL/kg)
<5 kg (n =15)	8.3 (2.6-10.9)	60 (30-200)	120 (0-2000)	2 (0-10)
5-10 kg (n = 14)	6.2 (2.5-9.3)	150 (30-200)	200(0-560)	2.6 (0-7)
10-20 kg (n = 5)	4.8 (4-5.7)	200 (150-800)	400(0-950)	1(0-3.5)
20-50 kg (n = 13)	3.7 (2-4.5)	540 (0-1400)	1100 (0-1700)	2.1 (0-6)
>50 kg (n = 3)	2 (2-3)	1800 (1000-2400)	2000 (2000-3500)	3 (1-3)
Total (n = 50)	5.2 (2-10.9)	150 (0-2400)	265 (0-3500)	2.4 (0-10)

*The results are given as average, minimum, and maximum values.

**Table 3:** Frequency of CRRT side effects.

Side effect	Patient (n)	%
Hypotension	28	56
Thrombocytopenia	24	48
Hypophosphatemia	22	44
Hypothermia	19	38
Hypokalemia	16	32
Bleeding	11	22
Hypocalcemia	10	20
Hypovolemia	3	6
Infection	2	4

### 3.1. Factors affecting outcomes and survival 

Fifty patients receiving CRRT had 6753 h of operation time with an 86% success rate for all patients. The most common problem in CRRT was the circuit changes due to occlusion in the circuits. CRRT terminated earlier than expected in 7 patients because of technical reasons (2 patients) and hemodynamic instability (5 patients). The overall survival rate was 42% and all survival-related factors are shown in Table 4. Survival rates in newborns were (64.2%), which was significantly higher when compared with others (P = 0.046) (Figure).

**Table 4:** Survival related factors.

	Survivors	Exitus	P-value
Demographic data			
Female	(n = 8) 16%	(n = 12) 24%	0.815
Male	(n = 1) 26%	(n = 17) 34 %	
Body weight (kg)	7.5	16.5	0.084
Age (month)	4	37	0.017
Intensive Care Monitoring			
PRISM score	20	29	0.016
PELOD score	17	31	0.001
Duration of hospitalization in PICU/NICU (days)	16.5	5	0.192
Biochemical data at the onset of CRRT			
BUN (mg/dL)	13	27	0.157
Creatinine (mg/dL)	1.03	1.26	0.120
Sodium (mEq/L)	137	138	0.679
Potassium (mEq/L)	3.7	4.3	0.032
Urine Output (ml/kg/h)	1.82	0.78	0.026
CRRT duration (hours)	72	72	0.992
CRRT modality			
CVVHF	(n=0)	(n= 2) 4%	0.321
CVVHD	(n= 7) 14%	(n= 5) 10%	
CVVHDF	(n= 14) 28%	(n= 22) 44%	
Vascular access location			
Femoral	(n= 5) 10%	(n= 13) 26%	0.277
Jugular	(n= 13) 26%	(n= 11) 22%	
Subclavian	(n= 3) 6%	(n= 4) 8%	
ECMO set	(n= 0)	(n= 1) 2%	
Catheter size	7	9	0.435
CRRT prescription			
Blood flow rate	6.4	4.4	0.02
Mean circuit lifespan (hours)	42	30	0.283

**Figure:**
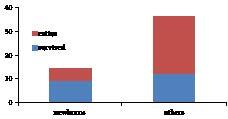
Survival rates of newborns and others.

The survival rates according to BW (<5 kg, 5-10 kg, 10-20 kg, 20-50 kg,> 50 kg) was evaluated and the highest survival rate 66.7% was achieved in infants less than 5 kg. However, there was no statistically significant difference between the weight groups (P = 0.190). The smallest survived patient was 3 kg. The highest treatment success was achieved in children with BW > 50 kg, but the success rates were not different between groups (P = 0.873). 

The highest survival rate was achieved in patients with primary renal disease (n = 3, 100%), and there was no survivor in the group of genetic disorders. All patients were also classified as those with a primary disease (n = 45) and those without (n = 5), and the survival rates were 40% and 60%, respectively. The presence of the primary disease has a negative effect on survival (P = 0.04). PRISM and PELOD scores were significantly different between the survivors and others (P = 0.016 vs. P = 0.001). There was no significant difference between survival rates and the length of hospitalization in PICU/NICU (P = 0.192).

Sodium, BUN, and creatinine levels were similar, but potassium levels and urine output were different between survivors and others. There was an inverse correlation between potassium level and survival (P = 0.032) and also the survival rate was better in patients with higher urine output (P = 0.026).

All patients received mechanical ventilator support. ECMO was administered to 8 patients and all have died. As a result, there was a statistically significant difference in survival between ECMO and the nonECMO group (P = 0.015). Plasma exchange was not found to be associated with survival (P = 0.979).

A patient with the diagnosis of theophylline intoxication was also survived. The highest survival rate was achieved in patients having just fluid overload (60%). There was no survivor among the patients treated with CRRT with the indication of electrolyte imbalance (hyperpotassemia, n = 2), the second-worst survival rate was among the acute kidney injury group (19%). There was no homogeneous distribution of mortality among the indications categories (P = 0.022) 

## 4. Discussion

Continuous renal replacement therapies are life-saving treatments for critically ill children and fluid burden, and removal of toxic substances and metabolites were restored. CRRT data are most widely obtained from the “prospective pediatric continuous renal replacement therapy” (ppCRRT) records [10]. The survival rate in our study is 42%, which is comparable with other single-center publications [11,12]. 

CRRT in young children with low-weights requires additional evaluation due to differences in extracorporeal circulating blood volume, high blood flow rates, and the presence of different underlying diseases and indications for this age group. Askenazi et al. [13] reported the results of children with a BW of ≤10 kg in the ppCRRT database, the overall survival rate was 43%, survival rates of the < 5 kg and 5-10 kg are 44% and 42%, respectively. They reported higher survival rates in children > 10 kg (64%) in the same database. Symons et al. [14] reviewed the records of 85 patients from 5 centers in the USA, with BW ≤10 kg, retrospectively. Overall survival was 38% and the survival rates of the < 3 kg and >3 kg are 25% (4 of 16 patients) and 41%, respectively. The smallest infant who was survived was 2.3 kg. In our study, conversely, the survival rate of newborns and BW <5 kg group seemed to be higher than the others (64.3% and 66.7%, respectively), but this is not statistically significant (P = 0.190). The lowest BW among the survivors is 3 kg. This condition may be due to the fact that the patients in the study group don’t distribute equally in all age groups and the most common CRRT indication in newborns is metabolic disease which is known to have better survival rates with CRRT treatment [15]. There were technical difficulties of CRRT for the patients with lower BW’s; however, a better survival rate was achieved in our center probably due to high quality technical and personnel support.

It is known that survival is associated with the primary disease in CRRT. According to the ppCRRT registry, survival rates were found to be 100% for drug intoxication, 84% for renal disease, 83% for tumor lysis syndrome, and 73% for congenital metabolic diseases [16]. In our study, the most common primary disease is metabolic disorders (24%). The group with the highest survival rate was renal disease group (100% survival in 3 patients). The percentage of the patients with a primary disease was 40% and this affects adversely the survival rate. Besides, 10% of the study group was organ transplanted patients (bone marrow, n = 4; liver, n = 1) and the survival rate among them was 40%, which is comparable to 45% survival rate in the study evaluating 51 patients requiring CRRT with stem cell transplantation [17].

In the ppCRRT registry, the most common indication for treatment is fluid overload with electrolyte imbalance. The highest achieved survival rate was 68% for electrolyte imbalance alone and the worst survival rate is in the group having electrolyte imbalance with fluid overload (51%) [10]. In another publication, patients with AKI due to different reasons treated with CRRT, the most common CRRT indication was fluid overload [18], also in our study, the most common indication for CRRT was AKI (40%) and the best survival rate is in the group of fluid overload (60%). However, due to the retrospective design of the study, the amount of fluid overload could not be determined. There was no survivor in the electrolyte imbalance group and the worst survival rate among other causes was found to be in the AKI group (20%).

Studies have shown that the PRISM II score is lower in the survivors [10,16]; however, this score is not a good predictor for children with AKI [19]. Goldstein et al. [20] reported that PRISM II scores recorded at admission to PICU were not different between the survivors and others, but it was higher at the beginning of CRRT and this may be associated with increased fluid burden. In our study group, the PRISM and PELOD scores recorded during the initial admission of patients were significantly lower in the survivors.

Also, high 24-h urine output (the day before the onset of CRRT) and low potassium levels found to have a positive effect on survival. This result may be related to better kidney functions and lower FO.

In the ppCRRT registries, CVVHD was applied to 48% of patients, CVVHDF 30%, CVVH 21%, and SCUF 1% [10], and the most common modality were CVVHD (60%) in the group of patients <10 kg, too [13]. In another study in pediatric patients, survival was higher when using the convective dialysis method (CVVH), but when metabolic disorders excluded, the significance disappeared [14]. In our study, CVVHD (72%) was the most frequent modality and there was no significant difference in survival between the diffusive and convective methods.

The effect of catheter entry site or size on CRRT performance in childhood remains unclear. In a study, catheter diameter has been reported to have a positive effect on the circuit lifespan [10]. There was no difference in catheter size or location between survivors and others in the ppCRRT registry [21]. Hackbarth et al. [22] showed that using larger diameter catheters, internal jugular veins for the access, and CVVHD application as factors will extend the circuit lifespan. To optimize circuit lifespan, the use of internal jugular vein in patients who need a <10 F catheter is advised. There was no difference in catheter size or catheter location between survivors and others in our study. The effect of catheter size and catheter location on circuit lifespan was not determined. 

Factors affecting the filter lifespan in patients with CRRT have not yet been fully determined. In a prospective study, the mean circuit lifespan was reported as 31 (1-293) h. There was no relationship between age, BW, diagnosis, vascular access site, blood flow rate, ultrafiltration rate, inotropic drug intake, and filter life. The use of heparin (20 U/kg/h), CVVHD application, filters with surface area 0.4-0.9 m², ≥6.5 F catheter size may prolong the filter life [23]. In another study, the effect of BW on circuit lifespan was questioned, and no difference was found in terms of circuit life between BW <5 kg and 5-10 kg [13]. The mean filter life was 39.1 h in our study, the longest circuit lifespan determined by using an M60 with a 7F catheter was 120 h in a patient whose BW is 20 kg. The shortest circuit lifespan life is 0.5 h in a 9-day newborn with metabolic disease. An HF20 circuit with an 8F catheter was used, and CRRT was terminated because of hemodynamic instability of the patient.

Although CRRT has many complications in critically ill children, adequate studies have not been conducted. In a prospective single-center study, hypotension was seen in 30.4% and hemorrhage in 10.3% at the onset of CRRT, and these were not related to other factors [24]. In our study, the most frequent side effect of CRRT was hypotension (56%) and rarest side effect was infection (4%). Hypotension may be related to hemodynamic instability of patients, which gets worse after CRRT. 

This article has limitations because of the retrospective design of the study. Descriptive information such as fluid overload amount and factors affecting circuit lifespan could not be obtained.   

In conclusion, CRRT is a lifesaving method in critically ill children with AKI and FO. In NICU and PICUs, CRRT can be performed in newborns and children at any age and weight by experienced teams. The success of this treatment depends on the patient’s clinical situation, underlying curable disease, controlling technical problems, and the experience of the teams. Eventually, CRRT is a very important extracorporeal treatment method and a lifesaving option in intensive care units. 

## Informed consent

This study was approved by the Ankara University Ethical Committee.
